# Dual Emergence of Usutu Virus in Common Blackbirds, Eastern France, 2015

**DOI:** 10.3201/eid2212.161272

**Published:** 2016-12

**Authors:** Sylvie Lecollinet, Yannick Blanchard, Christine Manson, Steeve Lowenski, Eve Laloy, Hélène Quenault, Fabrice Touzain, Pierrick Lucas, Cyril Eraud, Céline Bahuon, Stéphan Zientara, Cécile Beck, Anouk Decors

**Affiliations:** NSES Animal Health Laboratory of Maisons-Alfort, Maisons-Alfort, France (S. Lecollinet, S. Lowenski, C. Bahuon, S. Zientara, C. Beck);; ANSES Ploufragan, Ploufragan, France (Y. Blanchard, H. Quenault, F. Touzain, P. Lucas);; Departmental Veterinary Laboratory of Haut-Rhin (LVD68), Colmar, France (C. Manson);; ENVA, Maisons-Alfort (E. Laloy); ONCFS, Paris, France (C. Eraud, A. Decors)

**Keywords:** Usutu virus, flavivirus, emergence, blackbirds, France, zoonoses, viruses

**To the Editor:** Usutu virus (USUV) is a mosquitoborne flavivirus amplified in an enzootic cycle involving passeriform and strigiform birds as reservoir hosts and *Culex* mosquitos as vectors (*1*). Although originating from Africa, USUV has been introduced at least twice into central and western Europe, leading to substantial bird fatalities in central Europe (particularly in Austria, Hungary, Italy, Germany, and Switzerland) since 1996 (*2*). Its zoonotic potential has been recently highlighted in Italy in immunosuppressed patients who sought treatment for encephalitis (*3*).

Even though every country bordering France, apart from Luxembourg, has reported USUV in mosquitoes or wild birds recently, USUV outbreaks had not been reported in France, and only indirect evidence indicated circulation of USUV-like viruses in Eurasian magpies (*Pica pica*) in southeastern France (*4*). In 2015, the French event-based surveillance network SAGIR (*5*) reported increased fatalities of common blackbirds (*Turdus merula*) in 2 departments in eastern France, Haut-Rhin near the German border and Rhône (Figure). Five birds, 2 in Haut-Rhin and 3 in Rhône, were subjected to molecular detection for flaviviruses. During necropsy, their brains, hearts, livers, and kidneys (from 2 birds only) were sampled for RNA extraction and virus isolation. Tissues were homogenized in DMEM with ceramic beads (Qbiogen) and FastPrep ribolyzer (ThermoSavant). Total RNA was extracted with RNeasy kit (Qiagen) and flavivirus genomic RNA was amplified by conventional reverse transcription PCR with all of the tissues from 2 birds in Haut-Rhin that were found dead on August 5–10, 2015, and from 1 bird sampled on September 23 in Rhône (*6*). USUV was systematically identified in blackbird tissues by Sanger sequencing of the 1085-nt PCR fragment and BLAST analysis (https://blast.ncbi.nlm.nih.gov). Three USUV isolates were obtained after 2–3 passages in Vero cells, and whole-genome sequencing of every isolate was performed as previously described (*7*). Postmortem examination revealed hepatomegaly and splenomegaly in a USUV-infected blackbird and marked emaciation and kidney hemorrhages in another infected animal. A subset of samples was submitted for histologic analysis, but no microscopic lesions were found in any of the 3 USUV-positive blackbirds, suggesting that infection was hyperacute.

**Figure:**
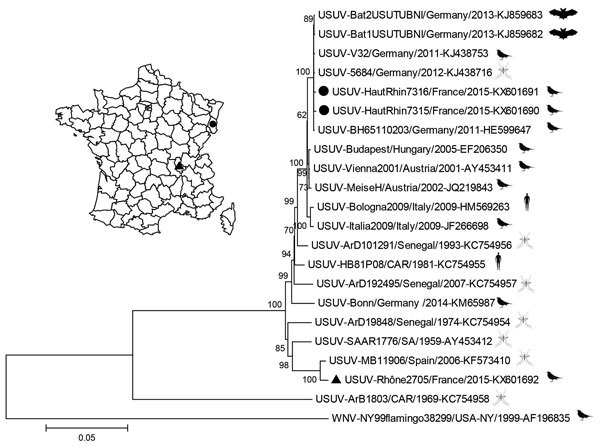
Phylogeny of Usutu virus (USUV) Haut-Rhin strains (black circles) and Rhône strain (black triangle), isolated in 2015 in eastern France compared with reference strains. Inset map shows locations where isolates were obtained. The strains from France are genetically distinct, with a homology of 95.7% at the nucleotide level and 98.8% (3,392–3 aa/3,434 aa) at the amino acid level. The evolutionary history was inferred by using the neighbor-joining method. The optimal tree with the sum of branch length 0.60224968 is shown. The percentage of replicate trees in which the associated taxa clustered together in the bootstrap test (1,000 replicates) are shown at the nodes. Evolutionary analyses were conducted in MEGA6 (http://www.megasoftware.net), and the evolutionary distances were computed by using the Jukes-Cantor method. The resulting tree is drawn to scale, with branch lengths in the units of number of base substitutions per site. The analysis involved 22 strains, including West Nile virus (WNV) as the root; GenBank accession numbers are indicated. All positions containing gaps and missing data were not included; 10,684 positions were included in the final dataset. An outline of the organism from which the virus was isolated (bat, bird, mosquito, or human) is placed next to the strain name. Scale bar indicates substitutions per site.

Phylogenetic analysis of the whole genome for the 3 USUV isolates demonstrated close genetic relatedness between USUV isolates from Haut-Rhin, France, and Germany (99.8% nucleotide identity with USUV-5684/Germany/2011, GenBank accession no. KJ438716) and between strains from Rhône, France, and Spain (99.2% identity with USUV-MB11906/Spain/2006, GenBank accession no. KF573410). Results showed that French USUV strains from Haut-Rhin and Rhône departments were clearly distinct from each other (95.7% nucleotide identity) and arose from >2 independent introduction events. In total, 41–42 nonsynonymous mutations were identified along the 3,434-aa long polyprotein, with capsid, nonstructural protein 2A, and nonstructural protein 4B having the highest nonsynonymous substitution rates of 96.0% (121/126), 97.4% (221/227), and 97.8% (311/318), respectively.

Symptomatic USUV infections were discovered in wild birds in France, indicating the emergence of USUV in counties in eastern France. Unusual and grouped bird fatalities observed in August and September 2015 in common blackbirds in Haut-Rhin and Rhône did not seem to alter blackbird population dynamics (data not shown). The viral strain recovered in Haut-Rhin, which borders Germany, is genetically similar to USUV strains isolated in central Europe, in particular in southwestern Germany in 2011. Such a finding further exemplifies the continuing and gradual diffusion of the Vienna USUV strain since 2001 (Austria in 2001, Hungary in 2005, Italy and Switzerland in 2006, Germany and Czech Republic in 2011, and Belgium in 2012) (*1*). The USUV strain isolated from the 1 blackbird in Rhône shared the highest genetic homology with USUV strains identified on 2 occasions in Spain: once in 2006 in Catalonia from *C. pipiens* mosquitoes and once in 2009 in Andalusia from *C. perexiguus* mosquitoes (*8*). 

Our findings indicate that the USUV/Spain strain can be pathogenic in birds. Symptomatic USUV infections in wild avifauna are difficult to quantify (because of low reporting rates and quick removal of dead birds by scavengers), and dynamic modeling of USUV in Austria indicated that a low proportion (0.2%) of USUV-killed birds had been effectively detected by USUV-specific surveillance programs (*9*). Mutations between USUV-Rhône2705/France/2015 and USUV-MB11906/Spain could also account for differential virulence in birds. These 2 strains differed by 14 nonsynonymous mutations (Technical Appendix Table). Although little is known about molecular determinants of USUV virulence, one can try to infer the importance of these mutations from data gained from studies on a closely related flavivirus, West Nile virus. In this respect, none of the 14 mutations observed have been found to be critical in flavivirus virulence.

Concomitantly with USUV emergence in France, another *Culex*-borne flavivirus, West Nile virus, has reemerged in southeastern France (*10*). Climatic and environmental conditions during the summer of 2015 seem to have promoted the spread of *Culex*-borne pathogens. However, risk factors for flavivirus emergence in France in 2015 have not been comprehensively analyzed.

Technical Appendix List of nonsynonymous mutations observed between USUV-Rhône2705/France/2015 and USUV-Spain/2006 (GenBank accession no. KF573410) and between USUV-HautRhin7315 or 7316/France/2015 and USUV-Germany/2012 (GenBank accession no. KJ438716).
