# Supplementary material for: Dual Emergence of Usutu Virus in Common Blackbirds, Eastern France, 2015
Source: Emerg Infect Dis. 2016 Dec;22(12):2225–7. doi: 10.3201/eid2212.161272 (PMC5189168; doi:10.3201/eid2212.161272)
Supplement: Technical Appendix — List of nonsynonymous mutations observed between USUV-Rhône2705/France/2015 and USUV-Spain/2006 (GenBank accession no. KF573410) and between USUV-HautRhin7315 or 7316/France/2015 and USUV-Germany/2012 (GenBank accession no. KJ438716). [file 16-1272-Techapp-s1.pdf]

DOI: http://dx.doi.org/10.3201/eid2212.161272

# Dual Emergence of Usutu Virus in Common Blackbirds, Eastern France, 2015

## Technical Appendix

**Technical Appendix Table.** Nonsynonymous mutations observed between USUV-Rhône2705/France/2015 and USUV-Spain/2006 (GenBank accession no. KF573410) and between USUV-HautRhin7315 or 7316/France/2015 and USUV-Germany/2012 (GenBank accession no. KJ438716). Nonsynonymous mutations were mainly reported in E and nonstructural proteins.

| Residue | USUV-Rhône2705/France/2015 | USUV-MB11906/Spain/2006 |
|---|---|---|
| M 147 | S | G |
| E 161 | A | V |
| E 343 | A | V |
| NS1 105 | T | A |
| NS1 269 | E | D |
| NS2A 1 | Y | H |
| NS2A 57 | C | Y |
| NS2A 119 | I | T |
| NS3 46 | F | L |
| NS3 268 | H | Y |
| NS3 288 | H | D |
| NS4B 122 | V | T |
| NS5 466 | H | Y |
| NS5 751 | A | T |
| Residue | USUV-HautRhin7315 or 7316/France/2015 | USUV-5684/Germany/2012 |
| E 126 | M | I |
| NS5 92 | S | P |
| NS5 117 | R | G |
